# Tailoring UV Penetration Depth in Photopolymer Nanocomposites: Advancing SLA 3D Printing Performance with Nanofillers

**DOI:** 10.3390/polym17010097

**Published:** 2025-01-01

**Authors:** Khalid Haj Ahmad, Zurina Mohamad, Zahid Iqbal Khan, Muddasar Habib

**Affiliations:** 1 College of Engineering, Alfaisal University, P.O. Box 50927, Riyadh 11533, Saudi Arabia; 2 Faculty of Chemical and Energy Engineering, Universiti Teknologi Malaysia, Johor Bahru 81310 UTM, Johor, Malaysia; 3 Department of Chemical Engineering, University of Engineering and Technology, Peshawar 25000, Pakistan; muddasarhabib@uetpeshawar.edu.pk

**Keywords:** photopolymer, nanocomposites, penetration depth, 3D printing

## Abstract

This study examines the influence of nanofillers on the ultraviolet (UV) penetration depth of photopolymer resins used in stereolithography (SLA) 3D printing, and their impact on printability. Three nanofillers, multiwalled carbon nanotubes (MWCNT), graphene nanoplatelets (xGNP), and boron nitride nanoparticles (BNNP), were incorporated into a commercially available photopolymer resin to prepare nanocomposite formulations. The UV penetration depth (Dp) was assessed using the Windowpane method, revealing a significant reduction with the addition of nanofillers. At a concentration of 0.25 wt.%, MWCNT showed the highest reduction in Dp (90%), followed by xGNP (65%) and BNNP (33%). SLA 3D printing was performed at varying nanofiller concentrations to evaluate printability. The findings highlight a strong correlation between Dp and the maximum printable nanofiller concentration, with MWCNT limiting printability to 0.05 wt.% due to its low Dp, while BNNP allowed printing up to 1.5 wt.%. Mechanical testing showed substantial improvements in hardness and elastic modulus, even at low nanofiller concentrations, with BNNP outperforming other fillers. Compared to a clear photopolymer, the elastic modulus for 3D printed nanocomposite samples with 0.05 wt.% nanofiller compositions showed an improvement of 43% for MWCNT, 63% for xGNP, and 104% for BNNP. The hardness results showed an improvement of 86% for MWCNT, 103% for xGNP, and 179% for BNNP. These results underscore the importance of Dp in determining the layer thickness and print success in SLA 3D printing. Practical applications include the design of advanced photopolymer nanocomposites for biomedical devices, electronics, and lightweight structural components. This research provides valuable insights for tailoring material properties to meet the demands of high-performance additive manufacturing.

## 1. Introduction

Three-dimensional printing, also known as 3D printing, is a versatile and rapidly growing field. It has a variety of applications in prototyping, jewelry, dentistry, pharmaceutical products, and customized manufacturing. It uses the layer-by-layer building of objects of any geometry, from a computer aided design source file. Its flexibility and reproducibility make 3D printing one of the most promising techniques for rapid prototyping and customized manufacturing.

Stereolithography is one of the most important 3D printing processes. It produces parts by polymerizing reactive resin using photoinitiators that are activated by the light source, which is a laser beam that moves to cure the layer being printed. This method can produce high-resolution parts with a good surface finish [1].

The curing behavior of photopolymers is characterized by the time required for network formation, gelation, and the establishment of the mechanical properties in the final product [2]. Cure depth is an important property of the photopolymer system, as it is vital to ensure adhesion between successive layers of the 3D printed part. It depends on the amount of energy provided by the light source, which can be controlled by the light intensity and the exposure time. The cure depth values are used to optimize the stereolithography (SLA) and digital light processing (DLP) printing parameters [3]. Penetration depth (D_p_) is important in determining the printability of photopolymer–nanofiller mixtures [4]. Nanofillers can be composed of a wide range of materials with improved properties and multifunctionality to 3D printing, such as electrical and thermal conductivities and mechanical strength [5]. Polymer nanocomposites show substantial property enhancements at a much lower content than polymer composites with conventional micron-scale fillers (such as glass or carbon fibers), which ultimately results in a lower component weight, and which can simplify processing. Moreover, the multifunctional property enhancements made possible with nanocomposites may create new applications for polymers [6].

While the purpose of adding nanofillers is to improve mechanical properties, some studies have showed that it leads to degradation, instead. The addition of graphene oxide to a commercially available acrylate-based photopolymer resulted in the lower elastic modulus and tensile strength of 3D printed samples [7,8]. The addition of hexagonal boron nitride nanoparticles to a commercially available acrylate-based photopolymer resulted in lower microhardness and compressive strength of 3D printed samples [9]. However, some studies showed that an improvement in the mechanical properties of a vat 3D printed nanocomposite could be achieved only with low concentrations of graphene, reaching up to 0.1 wt.% [10,11].

This study aims to investigate the impact of dispersing various nanofillers within photopolymer resins on their UV penetration depth, and the subsequent influence of this on the 3D printability of nanocomposites. By addressing the critical challenges in SLA 3D printing, such as optimizing layer adhesion, reducing defects, and enhancing mechanical properties, the study seeks to improve both the printability and functionality of photopolymer formulations. Through a systematic analysis of different nanofiller types and concentrations, this research provides detailed insights into the interplay between UV light penetration, light scattering, and absorption, which are key factors influencing the maximum printable layer thickness and mechanical performance of SLA-printed nanocomposites. This comprehensive approach bridges the gap between fundamental material properties and practical applications, offering tailored solutions for advanced additive manufacturing. The findings have significant implications for the design of high-performance photopolymer materials in diverse fields, including biomedical devices, lightweight structural components, and customized electronics.

## 2. Materials and Methods

### 2.1. Materials

The commercially available photopolymer resin Formlabs Clear (FLGPCL04) was obtained from Formlabs (Somerville, MA, USA). Multiwalled carbon nanotubes were purchased from Graphene Supermarket, USA. Boron nitride nanoparticles (BNNP), with an average particle size of 70 nm, were purchased from Sisco Research Laboratories (SRL) Pvt. Ltd., Mumbai, India. Three grades of graphene nanoplatelets (xGNP), namely C300, C500, and C750, with different surface areas, i.e., 300 m^2^/g, 500 m^2^/g, and 750 m^2^/g, respectively, were purchased from XG Sciences, Lansing, MI, USA.

### 2.2. Dispersion of Photopolymer Nanocomposites

Different photopolymer formulations, as shown in Table 1, were prepared by adding nanofiller into a photopolymer resin under shear mixing for 5 min, followed by sonication for 20 min. The mixture of photopolymer composites was used in penetration depth determination and in the 3D printing process.

### 2.3. Penetration Depth Determination

**Figure 1:**
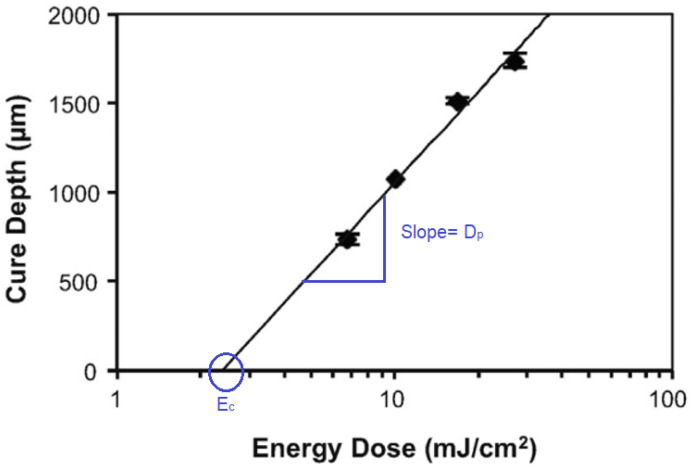
Plot of cure depth vs. energy dose example. Symbols: Cd represents the cure depth, E_0_ denotes the energy dose, and E_c_ indicates the critical exposure energy. The slope of the linear portion corresponds to the penetration depth (D_p_), essential for determining UV light interaction with the photopolymer (Source: [13]).

The working curve is used as the characterization method for any photopolymer system, as it provides information about penetration depth (D_p_) and critical energy dosage (E_c_). The curve is plotted based on experimental data, obtained from exposure time and cure depth. The graph is plotted with a logarithmic scale for energy dosage (E_0_) in the *x*-axis, and the corresponding cure depth (C_d_) in the *y*-axis. The critical exposure can be found from the intersection of the *x*-axis, and the penetration depth (D_p_) is found from the slope of the line, as shown in Figure 1 [3]. All values are presented as the mean derived from three to five independent sample tests, providing clarity and reliability in representing data variability.

The curing depth (C_d_) after the light exposure follows the Jacobs’ basic working curve equation [12]:

(1) $$C_{d}=D_{p}ln\;(\frac{E_{0}}{E_{c}})$$

**Figure 2:**
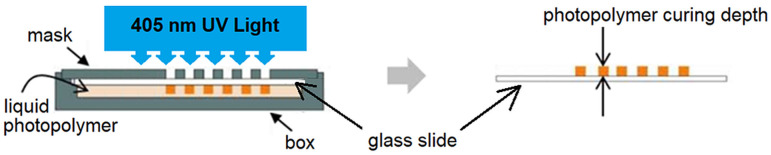
Setup for exposing photopolymer to UV light.

Figure 2 shows the Windowpane setup for the cure depth determination. Liquid photopolymer composites were first dispensed inside the box covered with the glass slide, below the mask that had small square windows, with the dimensions of 2 mm × 2 mm each, for ultraviolet (UV) light penetration. The photopolymer was then exposed to 405 nm UV light, chosen for its optimal absorption by the photoinitiators in the resin, ensuring efficient polymerization. The UV exposure was conducted using a FormCure unit (Formlabs, Somerville, MA, USA) at varying curing times (30, 60, 120, 240, and 480 s). A UV intensity of 10,000 Lux, which is equivalent to 1.46 mW/cm^2^, was used. The depth of the cured photopolymer and composites were measured using a micrometer. Table 2 shows the UV exposure energy for each time interval.

### 2.4. 3D Printing of Photopolymer Nanocomposites

Three formulations, clear photopolymer, BNNP (0.25 wt.%), and xGNP (C-500, 0.25 wt.%), were chosen to study the 3D printability of nanocomposites, based on their distinct UV penetration depths and mechanical performance characteristics. These selections were made to provide a representative comparison of different nanofiller types and concentrations.. The clear photopolymer, BNNP, xGNP (C-500), and MWCNT formulations were printed using a Form2 SLA (Formlabs, Somerville, MA, USA) printer at a 25-micrometer resolution. Two different post-curing methods were carried out for each composition, which is UV-cured at room temperature and UV-cured at 80 °C. FormCure unit (Formlabs, USA) was used for the UV-curing process.

### 2.5. Characterization of Printed Photopolymer and Nanocomposites

The Nanotest 3 nano-indentation platform (Micromaterials, Wrexham, UK) was used to determine the elastic modulus and hardness values of the printed samples. The load was measured as a function of the deformation depth. A Berkovich-type diamond indenter was used, with a maximum load of 20 mN. The experiment was performed in the following sequence: (1) approaching the surface, (2) loading to the peak load of 20 mN at a rate of 5 mN/s, (3) holding the indenter at peak load for 30 s, (4) unloading from the peak load at a rate of 20 mN/s. The holding step was included to eliminate the effect of creep. The elastic modulus and hardness values were obtained through the analysis using Nanotest software, version 3.0. Nanoindentation is widely used for determining the elastic modulus and hardness of polymeric materials [14].

## 3. Results and Discussion

### 3.1. UV Penetration Depth for Photopolymer and Its Nanocomposites

Working curves are widely used as a basic procedure for testing and characterizing photocurable resins [15]. The formulation for photopolymer-filled C-500 was chosen to demonstrate the working curve analysis. The cure depth was measured at different time intervals for 0.5 and 0.25 wt.% concentrations of C-500, and recorded in Table 3.

**Figure 3:**
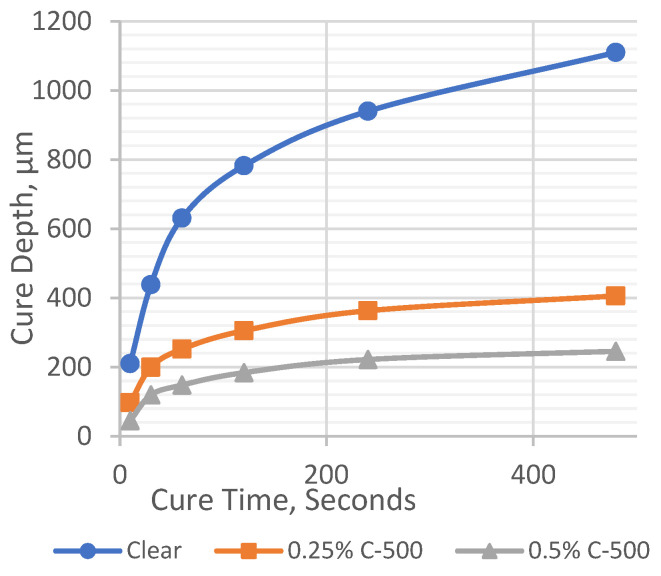
Cure time–cure depth curve.

**Figure 4:**
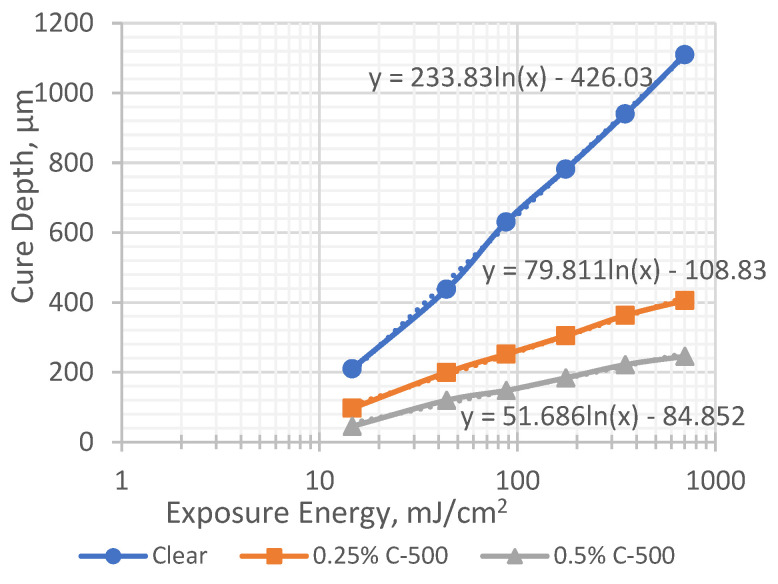
Working curve for clear and C-500 filled photopolymer.

Based on the data in Table 3, time versus cure depth (C_d_) values were plotted in Figure 3 for the clear photopolymer and photopolymer-filled C-500 at 0.25 wt.% and 0.5 wt.%. The resulting curves were converted to semi-log plots and the slope and intersection were found from the line equation of each plot, as shown in Figure 4 (working curve). The slope of the working curve equation in a semi-logarithmic graph represents the penetration depth, D_p_, and the intersection with abscissa critical represents the critical exposure energy E_c_ [16]. The working curves give information about the critical energy needed to start the crosslinking reaction and the time required to form a cured layer (Figure 4).

The cure depth (C_d_) increased with increasing the exposure time for the clear photopolymer. The penetration depth increased drastically until 120 s of cure time, and marginally increased after that. This is because the photoinitiator continues to absorb light after the reaction, which inhibits the penetration of light throughout the curing reaction, and reduces the UV penetration depth [17,18]. Introducing the nanofiller graphene C-500 drastically reduced the penetration depth of the photopolymer, and this was further reduced with the increased amount of the nanofiller. This is believed to be due to the hindering of UV light from penetrating through the filled photopolymer. Mitkus and Sinapius [4] reported that the dispersion of nanofillers results in a reduction in penetration depth, because nanofiller particles scatter the UV light and prevent deeper penetration. These data provide the information to allow the maximum printable layer thickness of different formulation to be printed using an SLA 3D printer.

**Figure 5:**
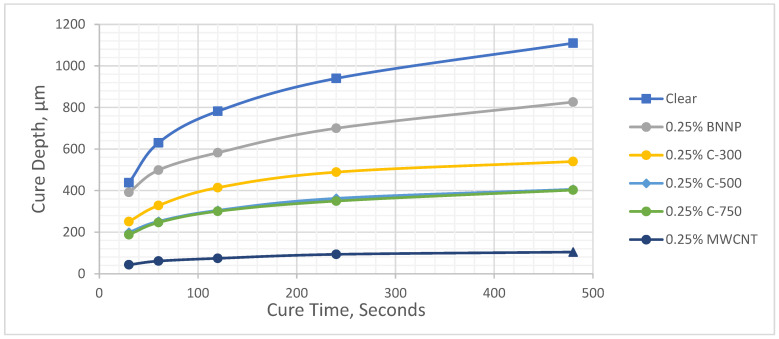
Cure depth versus cure time for 0.25 wt.% filler.

**Figure 6:**
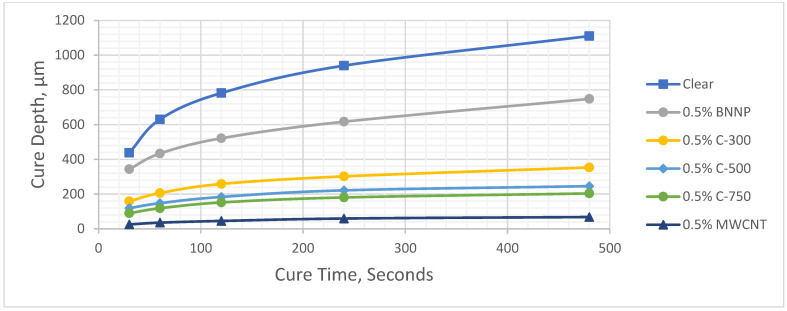
Cure depth versus cure time for 0.5 wt.% filler.

Figure 5 and Figure 6 show the cure depth versus cure time curves for different types of nanofiller-filled photopolymers at 0.25 wt.% and 0.5 wt.%, respectively. Eleven working curves were created for the clear photopolymer, and seven different nanofillers with two different compositions were created for each nanofiller. The determination coefficient, (R^2^), which indicates the proportion of variation, was above 0.98 for all working curves. The results are listed in Table 4 and Table 5.

**Figure 7:**
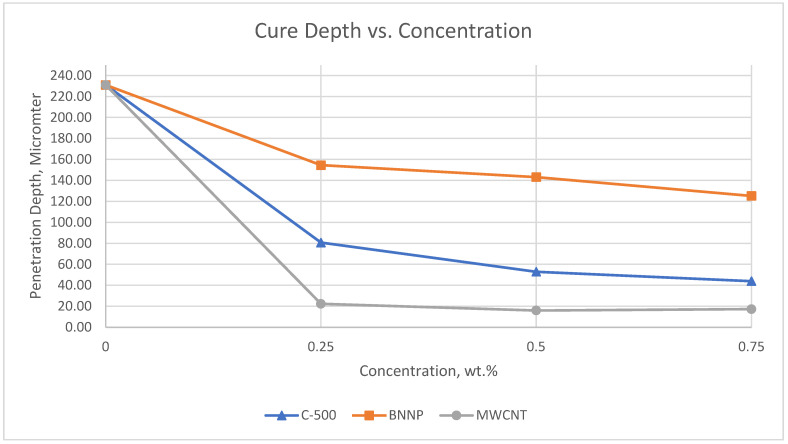
Cure depth versus concentration for three different nanofillers.

The results show that adding nanofillers (except for MWCNT) reduces the critical energy required to initiate polymerization reaction compared with a clear photopolymer. This can be attributed to the catalytic effect of nanofillers in the crosslinking reaction. The DSC thermograms in Figure 7 show that nanofillers have a hindering effect on curing reactions. This is evident from the larger area under the polymerization curve, which is larger for nanofiller mixtures compared with clear samples. This is consistent with penetration depth results, as the small area of clear sample indicates a higher degree of crosslinking. The addition of nanofillers results in shifting the exothermic peak from 163 °C to 153 °C for a 0.25 wt.% BNNP nanocomposite, and to 143 °C for a 0.25 wt.% xGNP nanocomposite. This can be attributed to the catalytic activity of these nanofillers. An analogous behavior has been reported in a previous study for curing phenolic resin, whose crosslinking peak shifts from 173 °C to 167 °C, due to an addition to the resin of two wt.% of graphene oxide [19]. Such an effect was also reported by previous studies for graphene-based materials [15,20].

The penetration depth (D_p_) decreased with the addition of nanofillers differently, based on the type and concentration of nanofiller. A previous study reported that UV penetration through photopolymer depends on factors like absorptivity and light scattering, which depends on the particle shape and size of the nanofiller [21]. From the results obtained, the D_p_ sequence is clear, for a >BNNP > C-300 > C-500/C-750 > MWCNT-filled photopolymer. Among these nanofillers, BNNP shows the highest D_p,_ and MWCNT causes the lowest D_p_. This is believed to be due to the differences in the scattering and absorptivity characteristics of different nanofillers, in addition to the homogeneity of nanofiller dispersion. This may impact the lower penetration depth of UV light, where the well-dispersed MWCNT block UV light and prevent penetration to longer distances through the photopolymer [4].

It can be noticed that the penetration depth of xGNP formulations depends on the surface area of the nanofiller. The sequence of the surface area from lower to higher values is C-300 < C-500 < C-750. Table 4 and Table 5 show that a lower xGNP surface area results in a higher penetration depth. This can be explained as a result of the better dispersion of the higher surface area xGNP, which leads to UV scattering. A previous study reported that the number of stacked layers dropped as the surface area of the xGNP fillers increased [22].

### 3.2. 3D Printed Photopolymer and Its Nanocomposites

Photopolymer nanocomposites of xGNP (C-500), BNNP, and MWCNT, at different nanofillers contents, were evaluated for printability using an SLA 3D printer. Different concentrations of each nanofiller, starting from 0.05 wt.%, were 3D printed at a 25-micrometer resolution to find the maximum printable concentration of different nanofillers. Experiments showed that MWCNT has very limited printable composition (0.05 wt.%) while it was possible to print a photopolymer with 0.25 wt.% C-500 and 1.5 wt.% BNNP. These results are consistent with the penetration depth results. At higher concentrations, the printing process failed due to the poor adhesion between successive layers, which may result from the reduced light penetration depth and decreased wetting of the cured surface by the liquid resin, potentially influenced by the presence of nanoparticles [23,24]. It can be observed from Figure 7 that the BNNP-filled photopolymer has the highest D_p_ values, while the MWCNT-filled photopolymer has the lowest values. These results give an indication that higher concentrations of BNNP-filled photopolymers can be 3D printed, compared to the other nanofillers. This may be attributed to the lower scattering of UV through the cured layer. Also, it can be observed that penetration depth drops to about 20 micron for 0.25 wt.% MWCNT. This clarifies the failure in printing process, as the penetration depth is smaller than the print layer thickness.

### 3.3. Mechanical Properties of 3D Printed Photopolymer Nanocomposites

**Figure 8:**
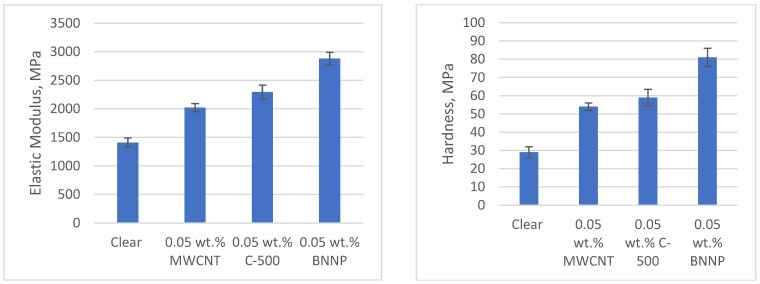
Elastic modulus and hardness of 0.05 wt.% composition nanocomposites.

Figure 8 shows the elastic modulus and hardness of photopolymer and nanocomposites of 0.05 wt.% nanofillers. The 3D nanocomposites were characterized mechanically, using nanoindentation. The elastic modulus and hardness of the as-printed samples improved in nanofillers compositions as low as 0.05 wt.%. This result is obvious for as-printed nanocomposite samples when compared with clear samples, as can be seen in Figure 8. Compared with the clear photopolymer, the elastic modulus for 3D printed nanocomposites with 0.05 wt.% nanofiller composition samples showed an improvement of 43% for MWCNT, 63% for xGNP, and 104% for BNNP. The hardness results showed an improvement of 86% for MWCNT, 103% for xGNP, and 179% for BNNP.

**Figure 9:**
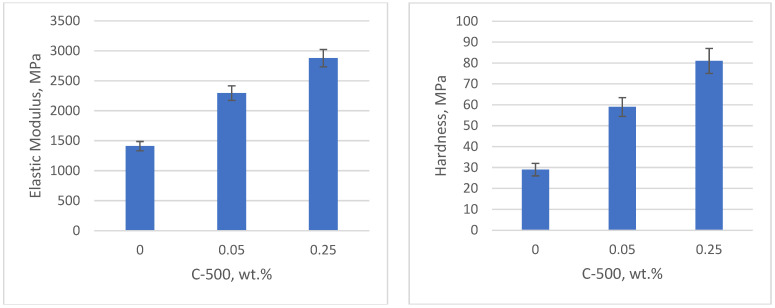
Elastic modulus and hardness of xGNP (C-500) nanocomposites.

**Figure 10:**
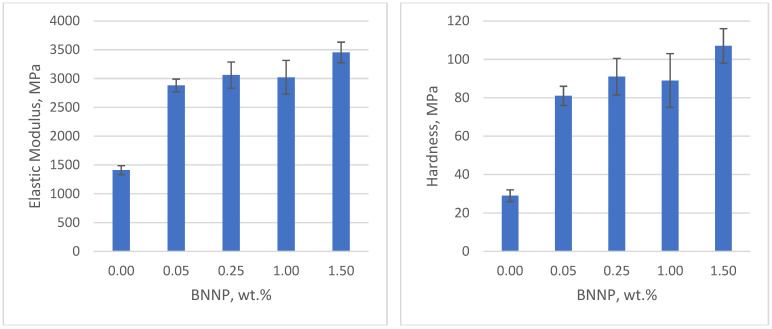
Elastic modulus and hardness of BNNP nanocomposites.

The effect of nanofiller concentration is depicted in Figure 9 and Figure 10. Figure 9 shows that increasing the C-500 content leads to an increase in mechanical properties in as-printed samples, with the highest elastic modulus and hardness observed at 0.25 wt.% C-500. Similarly, Figure 10 shows the improvement in BNNP-filled samples as the filler content increases. The printable composition in BNNP nanocomposites is higher than in C-500 nanocomposites. These results are in accordance with the penetration depth value of both nanofillers nanocomposites, where C-500 registered the lower values, compared with BNNP nanocomposites (Figure 7). A low cure depth and, thus, low printable compositions can be attributed to absorption, or the scattering of the light which prevents light from penetrating the photopolymer layer, with enough intensity to initiate the crosslinking reaction.

## 4. Conclusions

This study demonstrates that the addition of nanofillers significantly influences the UV penetration depth of photopolymer resins, which directly affects their printability in SLA 3D printing. Among the nanofillers tested, MWCNT caused the greatest reduction in penetration depth, while BNNP showed the least impact. These variations in penetration depth correlated strongly with the maximum printable nanofiller concentrations, where MWCNT allowed only 0.05 wt.% printability, compared to 1.5 wt.% for BNNP. The findings also highlight the interplay between light absorption, scattering effects, and nanofiller dispersion, which dictate the curing behavior and layer adhesion during printing. Additionally, mechanical characterization revealed notable improvements in hardness and elastic modulus with nanofiller incorporation, even at low concentrations, with BNNP exhibiting a superior performance. This work underscores the importance of tailoring nanofiller properties and concentrations to achieve the desired balance between printability and performance in SLA-printed nanocomposites. The insights gained pave the way for designing advanced photopolymer materials with enhanced functionality for diverse applications in additive manufacturing.

## 5. Future Scope

Future research could focus on optimizing photopolymer formulations with advanced or sustainable nanofillers to enhance printability and mechanical performance for industrial applications. Practical applications include the development of high-performance biomedical devices, such as dental implants and prosthetics, and customized electronic components with improved durability. Exploring eco-friendly nanofillers and refining 3D printing parameters, such as exposure times and layer thicknesses, can further improve efficiency and reliability. Post-processing techniques, such as heat treatment and UV curing, should be evaluated to enhance the mechanical and thermal stability of printed parts. Additionally, integrating these nanocomposites with emerging technologies, like 4D printing, and investigating their performance under real-world operational conditions will broaden their industrial relevance. These advancements align with sustainability goals, offering potential for scalable, environmentally friendly additive manufacturing solutions.

## Figures and Tables

**Table 1:** Formulations of photopolymer nanocomposites.

Nanofiller	Nanofiller Concentration (wt.%)
None	0
xGnP–C300	0.5
xGnP–C500	0.5
xGnP–C750	0.5
BNNP	0.5
MWCNT	0.5
xGnP–C300	0.25
xGnP–C500	0.25
xGnP–C750	0.25
BNNP	0.25
MWCNT	0.25

**Table 2:** Exposure times and energies used in cure depth determination.

Time, s	Energy, mJ/cm^2^
30	43.8
60	87.6
120	175.2
240	350.4
480	700.8

**Table 3:** Cure depth values for clear resin and photopolymer-filled C-500.

	Cure Depth, µm		
Time, s	Clear Resin	0.25 wt.% C-500	0.5 wt.% C-500
30	438	199.5	119.5
60	630.5	252	148
120	782	305	184
240	940	363	222
480	1110	406	246

**Table 4:** Penetration depth and critical exposure energy for 0.25 wt.% formulations.

	Penetration Depth, D_p_ (Micrometer)	% Reduction in D_p_	E_c_ (mJ/cm^2^)
Clear	231.00	-	5.87
0.25 wt.% BNNP	154.50	33.1	3.61
0.25 wt.% C-300	106.58	53.9	3.93
0.25 wt.% C-500	80.69	65.1	4.13
0.25 wt.% C-750	76.97	66.7	3.67
0.25 wt.% MWCNT	22.33	90.3	6.02

**Table 5:** Penetration depth and critical exposure energy for 0.5 wt.% formulations.

	Penetration Depth (Micrometer) D_p_	% Reduction in D_p_	E_c_ (mJ/cm^2^)
0.5 wt.% BNNP	143.12	38.0	4.23
0.5 wt.% C-300	70.04	69.7	4.53
0.5 wt.% C-500	52.88	77.1	5.75
0.5 wt.% C-750	41.84	81.9	4.95
0.5 wt.% MWCNT	15.98	93.1	9.21

## Data Availability

Currently, the raw data that are critical to reproducing these results are not available for public sharing, as they are an essential part of an ongoing doctoral project. The raw data will be confidential until the project is completed and published.
